# A comparative study of Neutrophil to Lymphocyte Ratio (NLR) in substance-free first episode of psychosis and substance-induced first episode of psychosis

**DOI:** 10.1192/j.eurpsy.2026.11615

**Published:** 2026-07-31

**Authors:** A. C. Matias, I. Marques, M. Bajouco

**Affiliations:** 1 Unidade Local de Saúde do Médio Tejo, Tomar; 2 Unidade Local de Saúde de Coimbra, Coimbra, Portugal

## Abstract

**Introduction:**

A phenotype characterized by increased inflammation has been associated with psychotic disorders. The complete blood count (CBC) with its related measurements, such as neutrophil to lymphocyte ratio (NLR), represent an easily obtainable biomarker that may assist in identifying this systemic inflammatory condition.

Examining biochemical indicators of inflammation in individuals experiencing a first episode of psychosis (FEP) could provide more precise insights into the inflammatory mechanisms underlying psychotic spectrum disorders.

**Objectives:**

This study aims to compare CBC inflammatory markers, particularly the NLR, in patients with substance-induced first episode of psychosis (SI-FEP) versus those with substance-free first episode of psychosis (SF-FEP).

**Methods:**

This retrospective study evaluated the CBC inflammatory markers, in this case NLR, in a cohort of 91 subjects with a FEP, 59 presented a SI-FEP and 32 a SF-FEP, recruited at UPEP (Unidade de Primeiro Episódio Psicótico), in Coimbra, Portugal.

**Results:**

There were no significant differences in NLR between the SI-FEP and SIP (t=-0,46; p=0,65).

**Conclusions:**

This study found no significant differences in inflammatory markers, specifically the neutrophil to lymphocyte ratio (NLR), between SI-FEP and SF-FEP patients. These results suggest that systemic inflammation, as measured by CBC markers, may not differ based on substance use status during the initial psychotic episode.

**Disclosure of Interest:**

None Declared

